# Sexual Networks of U.S. Military Service Members with Chlamydia at Joint Base San Antonio, June–December 2023

**Published:** 2026-01-26

**Authors:** Cecelia Peden, Scott A. Maddox, Tamico A. Stubblefield, Nancy S. Strahan, Yezenia Cadena-Malek, Cynthia L Bell, Eduardo MendezLanda, Joseph E. Marcus

**Affiliations:** School of Medicine, Uniformed Services University of the Health Sciences, Bethesda, MD: 2LT Peden; Army Public Health Nursing, Joint Base San Antonio–Fort Sam Houston, TX: Mr. Maddox, Ms. Stubblefield, Ms. Strahan, Ms. Cadena-Malek, Ms. Bell, CPT MendezLanda; Department of Medicine, Uniformed Services University of Health Sciences and Infectious Diseases Service, Brooke Army Medical Center, San Antonio, TX: Maj Marcus

## Abstract

Limited data on sexual networks in the U.S. military makes designing strategies to combat sexually transmitted infections (STIs) challenging. This retrospective evaluation assessed reported sexual networks of military service members with chlamydia, to inform future interventions for decreasing transmission of the infection. Thirty-two active duty service members at Joint Base San Antonio–Fort Sam Houston tested positive for chlamydia infection during the evaluation period, June through December 2023. Service members who tested positive for chlamydia were interviewed by Army Public Health Nursing staff and were asked to identify their sexual partners from the preceding 60 days, for routine contact tracing. Patient responses were then anonymized for comparisons of sexual networks of military service members—by sex, branch of service, and whether they were participating in military training or had completed training (“permanent party”). Service members with chlamydia were predominantly female (n=19, 59.4%), in the Army (n=18, 56.3%), and in military training (n=20, 62.5%). Of the 45 sexual contacts of the 32 service members identified through contact tracing, the majority (n=30, 66.7%) of those sexual contacts were civilians. Those still in military training were more likely to report sexual contacts who were also military service members, compared to permanent party service members (n=12, 50% vs. n=3, 14.3%, *p* =0.014). This evaluation determined that most service members who developed chlamydia were in sexual networks with only a single partner (n=22, 68.8%). These data should form an initial assessment of a military sexual network that needs to be confirmed in larger settings.

**What are the new findings?**

An analysis of sexual networks at Joint Base San Antonio–Fort Sam Houston involving 32 military service members with chlamydia found that sexual networks for service members who were in training had a greater proportion of sexual partners who were also in the military compared to service members who were not in training (50% vs. 14.3%, *p* =0.014).

**What is the impact on readiness and force health protection?**

In this population, the sexual networks of trainees diagnosed with chlamydia generally had a single partner, suggesting that broader testing strategies may be warranted to identify individuals who are at high risk for chlamydial infections.

Compared to civilian populations, U.S. military service members have had a greater burden of sexually transmitted infections (STIs). For example, the rate of new chlamydia infections for male service members ages 20-24 years is 1.5 times greater than their civilian peers. ^1 , 2^

Current U.S. Centers for Disease Control and Prevention (CDC) guidelines recommend annual screening for women under age 25 years to prevent complications of STIs such as pelvic inflammatory disease, in addition to consideration for screening of men and women ages 25 years and older who are in populations with high incidence of infection. ^3^ The U.S. military currently annually screens all women ages 25 years and younger, according to the CDC guidelines, and all services except the Army additionally screen women upon accession to military service. There is no universal screening program for men in the U.S. military.

The highest rates of chlamydia in the U.S. miliary are found in junior-enlisted women, and those age 24 years and younger. ^2^ Despite national U.S. military data suggesting a higher chlamydia burden among women, when universally screened, asymptomatic infections rates between male and female trainees are similar. ^4 , 5^ This underscores the need to evaluate increasing screening efforts among high-risk cohorts within the military.

Limited understanding of military sexual networks is a significant challenge for identifying high-risk cohorts within the military. Contact tracing serves as a useful epidemiological tool for uncovering such sexual networks, in addition to disrupting transmission events and re-infections. ^6^ This retrospective evaluation utilized Army Public Health Nursing (APHN) contact tracing data to identify the sexual networks of service members infected with chlamydia at a single military base, to inform future interventions.

## Methods

Active duty military service members who tested positive for chlamydia from June through December 2023 at Joint Base San Antonio–Fort Sam Houston were included in this evaluation of local sexual networks. Joint Base San Antonio–Fort Sam Houston supports 2 distinct groups: trainees and permanent party service members. Trainees, who are completing job-specific training following basic military training, live in congregate settings on base while fulfilling the requisite qualifications for their future military specialties. Trainees have restrictions on their abilities to physically leave their assigned military installations. Permanent party military service members, who have completed military training, have autonomy for their time off duty.

Military service members, regardless of training status, have universal access to no-cost medical care through Military Health System primary care, specialty, and emergency clinics. Women under age 25 years in the U.S. military are universally screened annually for chlamydia, while other populations are screened and tested based on symptoms and risk factors, as previously described. ^4^ All military service members are tested for chlamydia using the Aptima Combo 2 assay (Hologic, Marlborough, MA), and those who test positive are interviewed by a trained APHN nurse, for contact tracing to identify their sexual partners for the 60 days preceding diagnosis. In addition, those service members receive education on prevention of STIs and their re-infection.

In this retrospective evaluation of local sexual networks, the contact tracing results were anonymized to only specify the sex and training status of the source patient, and the sex and military status of their partner(s). Comparison of nominal variables between trainees and permanent party service members was performed with Fisher's Exact Test. A *p* -value of less than 0.05 was pre-determined to be significant.

Because this study involved analysis of de-identified, aggregate data only, it was classified as non-human subject research by the Brooke Army Medical Center Office of Human Research Protections Office (#23-17747) and consent was not required from participants.

## Results

Thirty-two active duty service members tested positive for chlamydia at Joint Base San Antonio–Fort Sam Houston during the study period, June through December 2023, and underwent contact tracing with APHN staff. The majority of service members who tested positive were female (n=19, 59.4%). Most service members who tested positive were in the Army (n=18, 56.3%), followed by the Navy (n=10, 31.3%), and Air Force (n=4, 12.5%). There were more trainees (n=20, 62.5%) than permanent party service members (n=12, 37.5%) among those who tested positive.

Ten male service members reported only female partners, 2 reported only male partners, and 1 reported male and female partners. All 19 female service members reported only male partners. The median number (interquartile range) of partners reported was 1 (1-2). Of the 45 partners identified through contact tracing, the majority (n=30, 66.7%) were not affiliated with the military.

Sexual networks differed by service member sex as well as training status **(Table)** . Both male trainees and permanent party members had majority female sexual partners (n=6, 85.7% and n=8, 72.7%, respectively) or who were civilians (n=5, 71.4% and n=9, 81.8%, respectively). Female permanent party members had predominantly civilian sexual partners (n=9, 90%) as well, while female trainees had majority military sexual partners (n=10, 58.8%). Service members in trainee status were more likely to have sexual contacts who were also military service members, when compared to permanent party service members (n=12, 50% vs. n=3, 14.3%, *p* =0.014).

**TABLE.** Sexual Networks of Military Service Members with Chlamydia, by Sex and Training Status

Source Patient		Total Partners	Median Partners	Military Partners ^a^						Civilian Partners ^a^					
				Total		Male		Female		Total		Male		Female	
	No.	No.	No. (IQR)	No.	%	No.	%	No.	%	No.	%	No.	%	No.	%
Total	32	45		15		12		3		30		19		11	
Males															
Permanent Party	7	11	1 (1-2)	2	18.2	1	9.1	1	9.1	9	81.8	2	18.2	7	63.6
Trainees	6	7	1 (1-1)	2	28.6	0	0.0	2	28.6	5	71.4	1	14.3	4	57.1
Females															
Permanent Party	5	10	2 (1-3)	1	10.0	1	10.0	0	0.0	9	90.0	9	90.0	0	0.0
Trainees	14	17	1 (1-2)	10	58.8	10	58.8	0	0.0	7	41.2	7	41.2	0	0.0

Abbreviation: IQR, interquartile range.

a Of total partners by sex and training status.

## Discussion

Despite elevated rates of chlamydia infection in military service members in comparison to their civilian counterparts, the sexual networks of the U.S. military population remain unknown. ^4^ In this evaluation cohort, most patients reported only 1 partner within the 60 days preceding diagnosis. Additionally, female trainees were more likely to have sexual partners who were also military service members.

Published data on sexual networks in U.S. military population are limited. ^7 - 9^ In 1 previously published study of 2,453 shipboard U.S. active duty Navy and Marine Corps personnel, 67% of most recent sexual partners were either service members or military beneficiaries, and among women this result increased to almost 80%. ^7^ In this retrospective evaluation, however, the majority of service members who tested positive for chlamydia had civilian partners. The findings of the prior study are consistent, however, with this evaluation's finding of a majority military affiliated sexual contacts among those in trainee status. In the earlier study, around 50% of women surveyed stated that they believed they had contracted an STI from a fellow service man, whereas 25% of service men stated that they believed they had contracted an STI from a service woman. ^7^ Those data are also congruent with this evaluation, suggesting that small sexual networks might facilitate transmission to both men and women, as seen in civilian populations. ^10^

Numerous studies have demonstrated multiple sexual partners as a risk for STI acquisition within the active duty military population. ^8 , 9 , 11^ Satterwhite et al. found, in the general U.S. population, regardless of sex, that reporting 2 to 4 sexual partners or 5 or more sexual partners within the past 12 months were significant predictors of reported STIs. ^12^ While this evaluation only investigated sexual networks from the preceding 60 days, the majority of active duty service members with chlamydia only reported 1 sexual partner. While this finding may represent under-reporting by service members to public health officers, or reflect the timing of contact tracing in relation to military duties, it is comparable to the number of sexual partners reported in other populations, such as college campuses. ^13^ Similarly, 2008 survey data of military women suggested that a majority (68.3%) had only 1 sexual partner in the last 12 months. ^11^

These data, taken together, may imply that certain high-risk populations have limited sexual networks. Unfortunately, limited sexual networks are harder to identify via contact tracing for an infection that is frequently asymptomatic. ^14^ Expanded or universal screening may be necessary to fully identify the STI burdens for such populations. Increased education efforts and prevention methods—such as condoms, which have demonstrated greater use by individuals with history of an STI, ^15^ as well as doxycycline post-exposure prophylaxis in populations that may benefit—may be useful strategies for military bases to consider. ^16 , 17^ Studies that demonstrate the effectiveness of specific STI prevention practices within military populations are currently lacking, however. ^18^

There are several limitations to this local evaluation of sexual networks. As a retrospective evaluation of anonymized records, the available data lack patient demographics, indications for testing or screening, setting of initial tests, and presence or absence of symptoms. An additional limitation is the small number of participants. Additionally, significant perceived stigma surrounds sexual health, and source patients are susceptible to reporting bias, potentially under-reporting their sexual contacts. ^19 , 20^ Notably, this project evaluated the sexual networks of service members who tested positive only for chlamydia, which may be different from sexual networks in relation to other STI transmission. Finally, as this sample is from 1 large military base, where the majority of testing is of female service members, ^21^ the external validity and extrapolation potential of these data are unknown.

Contact tracing provides insight into the sexual networks of military service members at a military base, which can inform more targeted local prevention and intervention strategies. From these data, it appears that limited sexual networks exist among military service members diagnosed with chlamydia, especially among trainees. These findings, from an initial evaluation at a single base, should be replicated in larger populations to produce more robust data, analyses, and findings for determining optimal prevention strategies throughout the U.S. military services.
